# Association between obesity indicators and retinopathy in US adults: NHANES 2005–2008

**DOI:** 10.3389/fnut.2025.1598240

**Published:** 2025-06-23

**Authors:** Chuan-Xi Wang, Miao Kuang, Jing-Jing Hou, Si-Yu Lin, Sheng-Zhen Liu, Ning Bao, Zheng-Xuan Jiang

**Affiliations:** ^1^Department of Ophthalmology, The Second Affiliated Hospital of Anhui Medical University, Hefei, China; ^2^Xuancheng Eye Hospital, Xuancheng, China; ^3^Department of Clinical Medicine, The Second School of Clinical Medicine, Anhui Medical University, Hefei, China

**Keywords:** obesity, retinopathy, NHANES, American, cross-sectional study

## Abstract

**Background:**

Obesity is harmful to the retina. Few studies have examined obesity indicators for retinopathy, and our research is intended to elucidate this relationship.

**Methods:**

This study assessed the association between obesity indicators and retinopathy using weighted multifactorial logistic regression, participants from the National Health and Nutrition Examination Survey (NHANES). The stability of the relationship was tested by subgroup analysis. Moreover, restricted cubic spline (RCS) was used to test for a non-linear relationship between them. Finally, Receiver Operating Characteristic (ROC) curves were applied to compare the prognostic value of each obesity indicator for retinopathy.

**Results:**

After adjusting the confounding factor, weighted logistic regression showed positive associations between body roundness index (BRI), conicity index (CI), waist-to-height ratio (WHtR), tri-ponderal mass index (TMI), weight-adjusted-waist index (WWI), body mass index (BMI), and retinopathy. RCS curves found linear correlations between BRI, CI, WHtR, TMI, and WWI with retinopathy, except for BMI. Subgroup analyses showed no significant interactions between age, sex, ethnicity, and education subgroups. ROC curves showed that WWI had the highest predictive value for retinopathy.

**Conclusion:**

Overall, increased levels of these obesity indicators increase the likelihood of developing retinopathy.

## 1 Introduction

Retinopathy is the most common ocular complication of diabetes mellitus (1), characterized by retinal microvascular dysfunction (2). It is thought to be the main reason for blindness in adults over 20 years in the United States (3). Previous research found that patients with retinopathy were more likely to suffer from heart failure, stroke, kidney disease (4), and neurological disorders. Retinopathy may be a marker for the risk of reactive vascular disease (5). This shows that retinopathy affects not only vision but also other vascular complications. Therefore, identifying the risk factors that influence retinopathy is crucial for systemic microvascular health.

Obesity and diabetes are well-established risk factors for retinal diseases (6, 7). Enhanced oxidative stress induced by diabetes plays a central role in the pathogenesis of retinopathy diseases by contributing to retinal cell dysfunction and apoptosis (8). It also triggers the release of pro-inflammatory cytokines, which can lead to basement membrane thickening, disruption of the blood-retinal barrier (BRB), microvascular occlusion, and ultimately, the development of retinopathy (9, 10).

Obesity is a significant public health problem worldwide (11), and the incidence of overweight and obesity has continued to increase in recent years (12). Obesity is also closely linked to retinal diseases, potentially contributing through mechanisms such as insulin resistance, systemic inflammation, oxidative stress, and microvascular dysfunction (13, 14). Although body mass index (BMI) is widely used to assess obesity, it does not accurately reflect fat distribution. Abnormal fat accumulation can occur even in individuals with a normal BMI (15). While imaging techniques such as computerized tomography (CT) and magnetic resonance imaging (MRI) can directly quantify visceral adiposity, their highly cost limits large-scale clinical use (16). Therefore, several alternative anthropometric indices have been developed to provide low-cost, practical assessments of body fat distribution: body roundness index (BRI), conicity index (CI), waist-to-height ratio (WHtR), tri-ponderal mass index (TMI), weight-adjusted-waist index (WWI), and a body shape index (ABSI).

BRI based on eccentricity theory and incorporating waist circumference and height, offers a more nuanced evaluation of visceral fat and abdominal obesity (17, 18). The WWI, introduced by Park et al., is calculated as waist circumference divided by the square root of body weight, enabling the assessment of central obesity independent of total body weight (19). The WHtR is another simple yet effective index that reflects central fat distribution (20). Additionally, the ABSI and the CI are commonly used to evaluate fat distribution, with ABSI more indicative of central obesity and CI focusing specifically on abdominal adiposity (21).

Previous studies have demonstrated that: compared to general obesity, central obesity is more strongly associated with insulin resistance and diabetes mellitus—both of which are key contributors to retinal diseases (22). While much research has been conducted on the association between these anthropometric indices and conditions such as diabetes, hypertension, and kidney stones (23–25), the relationship between these obesity-related measures and retinal diseases remains unclear. Additionally, it is not known which indicator has the greatest diagnostic value for retinal diseases. Therefore, we used data from the National Health and Nutrition Examination Survey (NHANES) from 2005 to 2008 to examine the relationship between various measures and the prevalence of retinal diseases in the U. S. population. We also compared the diagnostic value of novel and traditional indicators for retinal diseases using the receiver operating characteristic (ROC) method.

## 2 Methods

### 2.1 Research population

This study used publicly available data from the NHANES from two consecutive cycles, 2005–2006 and 2007–2008. The NHANES is a cross-sectional survey conducted by the US National Institutes of Health to assess the health and nutritional status of the US population.

**Figure 1:**
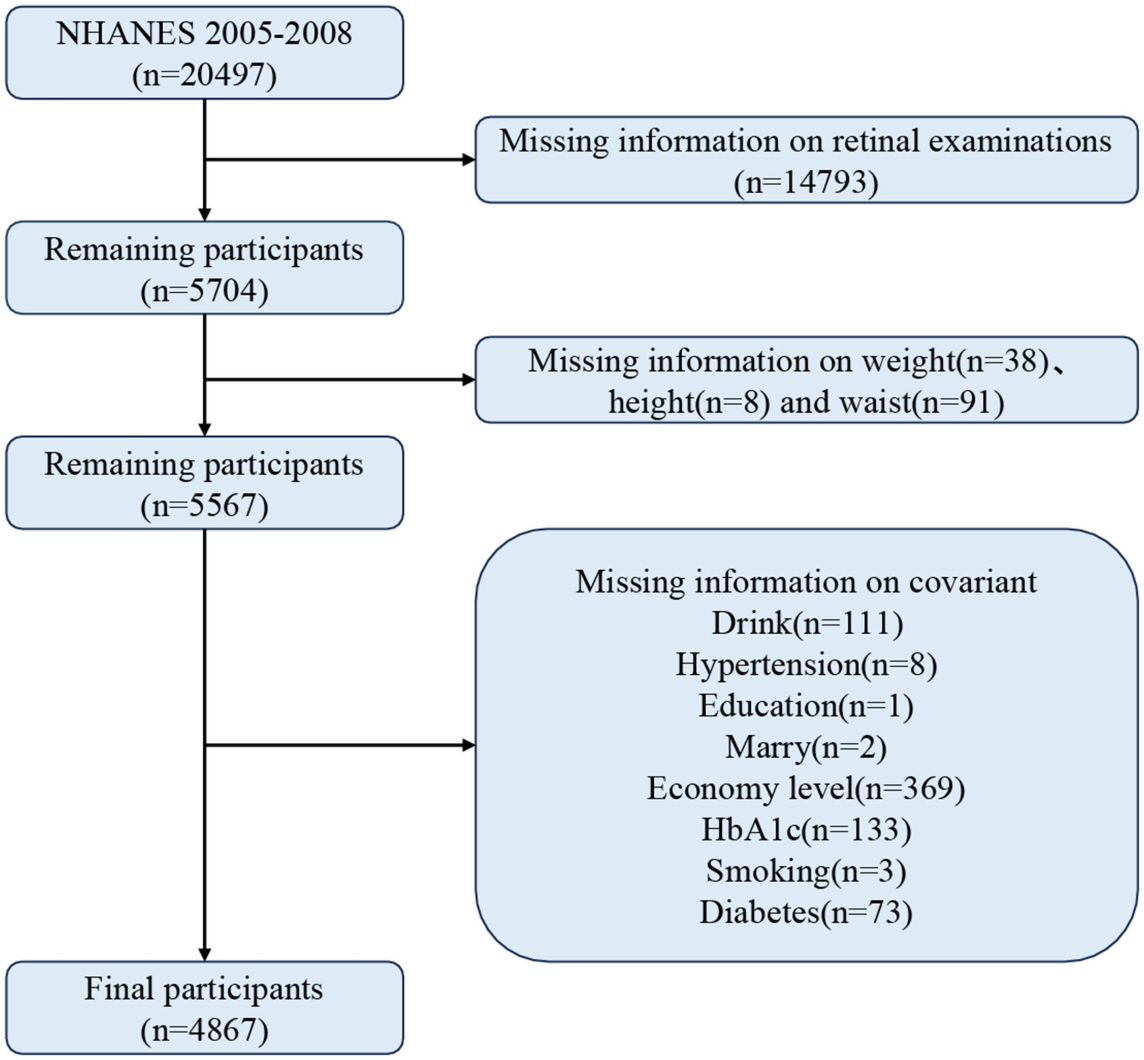
Screening flowchart for the study.

There were 20,497 participants in this study, and participants who did not meet the following criteria were excluded: (1) missing data on retinal examination, (2) missing information on waist circumference, height, and weight, and (3) missing data on relevant covariates. In the end, 4,867 participants met the criteria. Figure 1 demonstrates the detailed screening process.

### 2.2 Assessment of retinopathy

Technicians used a Canon fundus camera to take color photographs of participants’ retinas, two from each eye. Two professional graders graded pictures according to the Early Treatment Diabetic Retinopathy Study (ETDRS) criteria: no retinopathy (<14 grades), retinopathy (> = 14 grades).

### 2.3 Calculation of obesity indicators

We calculate the following indicators using waist, height, and weight. The unit of weight is “kg,” the unit of height is “m,” and the unit of the waist is “m,” except for WWI.

$$\text{ABSI}=\text{waist}/{\left(\text{height}\right)}^{1/2*}{\left(\mathrm{BMI}\right)}^{2/3}$$

$$\mathrm{BRI}=364.2-365.5^{*}{\left\{1-[{\left(\text{waist}/2\pi \right)}^{2}/{\left(\text{height}/2\right)}^{2}]\right\}}^{1/2}$$

WWI=waist/(weight)1/2

WHtR=waist/height

$$\mathrm{CI}=\text{waist}/[0.109^{*}{\left(\text{weight}/\text{height}\right)}^{1/2}]$$

$$\mathrm{TMI}=\text{weight}/{\left(\text{height}\right)}^{3}$$

$$\mathrm{BMI}=\text{weight}/{\left(\text{height}\right)}^{2}$$

### 2.4 Assessment of covariates

To better assess the relationship between these obesity indicators and retinopathy, we included influential covariates: age, sex, ethnicity, education, poverty income ratio, marital status, hemoglobinA1c (HbA1c), smoking and drinking status, and whether they had hypertension and diabetes. Of these, smoking, alcohol consumption, and hypertension were obtained from self-reported questionnaires. Diabetes was diagnosed if one of the following criteria was met: (1) being told by a doctor that they had diabetes; (2) taking glucose-lowering medication or insulin; and (3) having a HbA1c ≥ 6.5%.

### 2.5 Statistical analysis

Participants were divided into groups with and without retinopathy according to whether they had retinopathy. Continuous variables were expressed as weighted means (standard error) and categorical variables as sample sizes (weighted percentages), and statistical discrepancies between the different clusters were assessed using the *t*-test and Rao-Scott *χ*, respectively. We used weighted logistic regressions to test the association of obesity markers with retinopathy in the US and diabetic populations, respectively. Restricted cubic spline (RCS) models were applied further to detect a non-linear connection between obesity markers and retinopathy. In addition, we performed subgroup analyses and calculated interaction *p*-values according to age, sex, race, and education to assess the stability of their relationships. Finally, we calculated the area under the curve based on ROC curves to compare the predictive value of each indicator for retinopathy. Statistical analysis for this investigation was conducted using R (version 4.2.1) and EmpowerStats (version 2.0), with *p* < 0.05 as significant.

## 3 Results

### 3.1 Characteristics of participants

A total of 4,867 participants were enrolled, 47.6% of whom were male, with an average age of 56.18 (0.40). Of these participants, 596 were diagnosed with retinal disease. Table 1 shows more detailed information. People with retinopathy were more likely to be male, non-Hispanic White, educated to college level and above than participants without retinopathy, and to have higher levels of ABSI, BRI, CI, WHtR, TMI, WWI, and BMI. Retinal scores also divided the retinopathy group into a non-proliferative retinopathy group (grades 14–51) and a proliferative retinopathy group (grades 60–80). The results showed higher levels of ABSI, BRI, CI, WHtR, TMI, WWI, and BMI in the proliferative retinopathy group compared to the other.

**Table 1:** Demographics classified with retinopathy.

Characteristics	All	Non-retinopathy	Retinopathy				
			NPR + PR	*p*-value	NPR	PR	*p*-value
Number	4,867	4,271	596		575	21	
Gender (*N*, %)				<0.001			
Male	2,441 (47.6)	2099 (46.4)	342 (57.1)		333 (57.0)	9 (61.6)	<0.001
Female	2,426 (52.4)	2,172 (53.6)	254 (42.9)		242 (43.0)	12 (38.4)	
Age [years, mean (SE)]	56.18 (0.40)	55.80 (0.28)	59.77 (0.38)	<0.001	59.72 (0.54)	61.75 (0.47)	<0.001
Ethnicity (*N*, %)				<0.001			<0.001
Mexican American	743 (5.3)	636 (5.1)	107 (7.0)		102 (6.9)	5 (10.0)	
Other Hispanic	322 (3.0)	281 (2.9)	41 (4.1)		36 (4.0)	5 (9.2)	
Non-Hispanic White	2,682 (78.1)	2,426 (79.1)	256 (69.0)		252 (69.3)	4 (58.1)	
Non-Hispanic Black	966 (9.1)	791 (8.4)	175 (15.4)		168 (15.2)	7 (22.6)	
Other	154 (4.5)	137 (4.5)	17 (4.5)		17 (4.6)	0 (0.0)	
Education (*N*, %)				<0.001			<0.001
Less Than 9th Grade	635 (6.3)	521 (5.9)	114 (10.7)		107 (10.0)	7 (39.7)	
9–11th Grade	737 (11.1)	626 (10.7)	111 (14.4)		106 (14.4)	5 (10.9)	
High school graduate or equivalent	1,208 (26.2)	1,055 (25.7)	153 (31.4)		151 (32.0)	2 (3.9)	
Some College or AA degree	1,251 (28.4)	1,108 (28.5)	143 (27.0)		138 (27.1)	5 (24.3)	
College graduate or above	1,036 (28.0)	961 (29.1)	75 (16.6)		73 (16.5)	2 (21.1)	
Economic level (*N*, %)				<0.001			<0.001
<1	747 (8.9)	648 (8.8)	99 (9.7)		91 (9.3)	8 (27.4)	
1–3	2026 (33.3)	1733 (32.1)	293 (44.5)		282 (43.9)	11 (68.7)	
>3	2094 (57.9)	1890 (59.1)	204 (45.8)		201 (46.8)	2 (3.9)	
Marital status (*N*, %)				0.769			0.489
Unmarried or other	1730 (30.3)	1,512 (30.2)	218 (30.9)		206 (30.6)	12 (43.9)	
Married or living with a partner	3,137 (69.7)	2,759 (69.8)	378 (69.1)		369 (69.4)	9 (56.1)	
Alcohol consumption (*N*, %)				0.009			0.010
Yes	3,362 (73.1)	2,981 (73.8)	381 (66.6)		368 (66.4)	13 (75.3)	
No	1,505 (26.9)	1,290 (26.2)	215 (33.4)		207 (33.6)	8 (24.7)	
Smoking status (*N*, %)				0.202			0.204
Never	2,296 (48.5)	2015 (48.6)	281 (46.9)		268 (46.5)	13 (62.3)	
Now	988 (20.6)	870 (20.2)	118 (23.9)		117 (24.4)	1 (3.2)	
Former	1,583 (31.0)	1,386 (31.2)	197 (29.3)		190 (29.1)	7 (34.6)	
Hypertension (*N*, %)				<0.001			<0.001
Yes	2,214 (41.1)	1857 (40.0)	357 (51.9)		340 (50.9)	17 (93.4)	
No	2,653 (58.9)	2,414 (60.0)	239 (48.1)		235 (49.1)	4 (6.6)	
Diabetes (*N*, %)				<0.001			<0.001
Yes	940 (13.9)	651 (11.1)	289 (40.1)		268 (38.7)	21 (100.0)	
No	3,927 (86.1)	3,620 (88.9)	307 (59.9)		307 (61.3)	0 (0.0)	
HbA1c (%)	5.66 (0.90)	5.58 (0.74)	6.46 (1.64)	<0.001	6.42 (1.60)	8.22 (2.23)	<0.001
ABSI [mean (SE)]	0.08 (0.00)	0.08 (0.00)	0.08 (0.00)	0.001	0.08 (0.00)	0.09 (0.00)	<0.001
WtHR [mean (SE)]	0.60 (0.00)	0.59 (0.00)	0.62 (0.00)	<0.001	0.62 (0.00)	0.66 (0.00)	<0.001
CI [mean (SE)]	1.32 (0.00)	1.32 (0.00)	1.34 (0.00)	<0.001	1.34 (0.00)	1.41 (0.00)	<0.001
BRI [mean (SE)]	5.50 (0.06)	5.45 (0.05)	5.97 (0.10)	<0.001	5.95 (0.09)	6.85 (0.07)	<0.001
WWI [mean (SE)]	11.08 (0.02)	11.05 (0.02)	11.28 (0.01)	<0.001	11.27 (0.03)	11.78 (0.01)	<0.001
BMI [mean (SE)]	29.06 (0.15)	28.97 (0.13)	29.94 (0.15)	0.005	29.91 (0.16)	31.10 (0.12)	0.018
TMI [mean (SE)]	17.29 (0.09)	17.23 (0.10)	17.82 (0.09)	0.005	17.82 (0.08)	18.23 (0.07)	0.017

PR, proliferative retinopathy; NPR, non-proliferative retinopathy; SE, standard error; ABSI, A Body Shape Index; BMI, Body Mass Index; BRI, Body Roundness Index; CI, Conicity Index; WHtR, Waist-to-Height Ratio; TMI, Tri-ponderal Mass Index; WWI, Weight-adjusted-Waist Index.

### 3.2 Relationship between obesity indicators and retinopathy in the American population and the diabetic population

Multifactorial weighted logistic regression showed that WHtR, CI, BRI, WWI, BMI, and TMI were positively associated with retinopathy in the total population. When converted to categorical variables by quartile, compared to Q1 participants, Q4, the risk of retinopathy increased by 65, 44, 65, 56, 64, and 84%, respectively. In the diabetic population, we found a negative association between WWI and retinopathy, with a 39% decreased risk of having retinopathy in group Q3 (11.619–12.065) compared with group Q1 (9.381–11.136). The other indicators of obesity were not statistically significant. Detailed information can be found in Table 2 and Supplementary Table S1.

**Table 2:** Relationship between new obesity indicators and retinopathy in the American population and the diabetic population.

Variable	American population				Diabetes population			
	Model 1		Model 2		Model 1		Model 2	
	OR (95%CI)	*p-*value	OR (95%CI)	*p-*value	OR (95%CI)	*p-*value	OR (95%CI)	*p-*value
ABSI								
Continuous	5.82 (8.06, 41.95)	0.002	1.23 (0.94, 1.59)	0.445	>100 (0.00, >100)	0.594	89.65 (0.00, >100)	0.611
Categories								
Q1	ref		ref		ref		ref	
Q2	1.11 (0.72, 1.70)	0.653	0.95 (0.59, 1.51)	0.817	0.90 (0.54, 1.50)	0.689	0.83 (0.50, 1.38)	0.489
Q3	1.48 (0.95, 2.31)	0.094	1.07 (0.64, 1.80)	0.794	0.89 (0.57, 1.38)	0.608	0.79 (0.50, 1.24)	0.321
Q4	1.70 (1.09, 2.66)	0.026	1.04 (0.62, 1.73)	0.893	0.89 (0.57, 1.38)	0.456	0.96 (0.57, 1.61)	0.882
WHtR								
Continuous	13.97 (4.52, 43.18)	<0.001	6.97 (1.85, 26.35)	**0.007**	0.17 (0.02, 1.61)	0.132	0.26 (0.02, 3.63)	0.293
Categories								
Q1	ref		ref		ref		ref	
Q2	1.18 (0.78, 1.79)	0.434	1.02 (0.69, 1.52)	0.908	0.71 (0.42, 1.19)	0.204	0.78 (0.48, 1.28)	0.347
Q3	1.48 (0.93, 2.37)	0.108	1.18 (0.76, 1.83)	0.476	0.78 (0.46, 1.29)	0.337	0.82 (0.48, 1.40)	0.478
Q4	2.03 (1.52, 2.70)	<0.001	1.65 (1.22, 2.24)	**0.007**	0.64 (0.38, 1.07)	0.098	0.75 (0.44, 1.27)	0.306
CI								
Continuous	30.05 (6.41, 140.87)	<0.001	5.15 (1.04, 25.61)	**0.046**	0.49 (0.04, 6.03)	0.584	0.27 (0.02, 3.56)	0.292
Categories								
Q1	ref		ref		ref		ref	
Q2	1.59 (1.17, 2.18)	0.007	1.31 (0.96, 1.79)	0.114	0.60 (0.37, 0.95)	0.039	0.61 (0.38, 1.09)	0.067
Q3	1.62 (1.12, 2.36)	0.017	1.19 (0.82, 1.71)	0.376	0.71 (0.43, 1.16)	0.180	0.62 (0.38, 1.03)	0.092
Q4	2.28 (1.61, 3.24)	<0.001	1.44 (1.04, 1.99)	**0.041**	0.75 (0.45, 1.24)	0.264	0.65 (0.40, 1.06)	0.112
BRI								
Continuous	1.11 (1.06, 1.16)	<0.001	1.08 (1.03, 1.14)	**0.008**	0.94 (0.86, 1.02)	0.162	0.95 (0.87, 1.05)	0.345
Categories								
Q1	ref		ref		ref		ref	
Q2	1.18 (0.78, 1.79)	0.434	1.02 (0.69, 1.52)	0.908	0.71 (0.42, 1.19)	0.204	0.78 (0.48, 1.28)	0.347
Q3	1.48 (0.93, 2.37)	0.108	1.18 (0.76, 1.83)	0.476	0.78 (0.46, 1.29)	0.337	0.82 (0.48, 1.40)	0.478
Q4	2.03 (1.52, 2.70)	<0.001	1.65 (1.22, 2.24)	**0.007**	0.64 (0.38, 1.07)	0.098	0.75 (0.44, 1.27)	0.306
WWI								
Continuous	1.48 (1.22, 1.80)	<0.001	1.25 (1.03, 1.53)	**<0.030**	0.90 (0.69, 1.17)	0.441	0.91 (0.69, 1.22)	0.515
Categories								
Q1	ref		ref		ref		ref	
Q2	1.35 (0.94, 1.94)	0.122	1.14 (0.79, 1.63)	0.506	0.73 (0.49, 1.08)	0.131	0.76 (0.52, 1.12)	0.194
Q3	1.56 (1.11, 2.21)	0.017	1.19 (0.84, 1.70)	0.343	0.61 (0.39, 0.95)	0.036	0.61 (0.39, 0.97)	**0.041**
Q4	2.28 (1.64, 3.17)	<0.001	1.56 (1.16, 2.10)	**0.012**	0.85 (0.53, 1.36)	0.499	0.86 (0.53, 1.40)	0.558
BMI								
Continuous	1.02 (1.01, 1.04)	0.004	1.02 (1.00, 1.04)	**0.022**	0.98 (0.95, 1.00)	0.085	0.98 (0.95, 1.01)	0.211
Categories								
Q1	ref		ref		ref		ref	
Q2	1.28 (0.82, 2.00)	0.281	1.22 (0.79, 1.89)	0.395	1.05 (0.60, 1.84)	0.873	1.00 (0.56, 1.80)	0.994
Q3	1.60 (1.15, 2.24)	0.010	1.45 (1.05, 2.01)	**0.046**	0.76 (0.48, 1.21)	0.258	0.75 (0.46, 1.21)	0.261
Q4	1.70 (1.23, 2.33)	0.003	1.64 (1.15, 2.33)	**0.018**	0.68 (0.40, 1.14)	0.155	0.75 (0.43, 1.32)	0.334
TMI								
Continuous	1.04 (1.01, 1.06)	0.004	1.04 (1.01, 1.07)	**0.016**	0.96 (0.92, 0.99)	0.049	0.97 (0.92, 1.02)	0.264
Categories								
Q1	ref		ref		ref		ref	
Q2	1.27 (0.89, 1.83)	0.203	1.28 (0.91, 1.81)	0.179	0.82 (0.51, 1.32)	0.419	0.79 (0.48, 1.28)	0.352
Q3	1.31 (0.98, 1.74)	0.079	1.21 (0.90, 1.64)	0.235	0.70 (0.47, 1.05)	0.099	0.69 (0.43, 1.10)	0.141
Q4	1.78 (1.37, 2.33)	<0.001	1.84 (1.35, 2.52)	**0.002**	0.65 (0.41, 1.04)	0.081	0.79 (0.47, 1.33)	0.390

Model 1: unadjusted.

Model 2: sex, age, ethnicity, educational level, marital status, economic level, alcohol consumption, smoking status, and hypertension.

Continuous variables were converted to categorical variables according to the quartile method, as detailed in Supplementary Table S1.

ABSI, A Body Shape Index; BMI, Body Mass Index; BRI, Body Roundness Index; CI, Conicity Index; WHtR, Waist-to-Height Ratio; TMI, Tri-ponderal Mass Index; WWI, Weight-adjusted-Waist Index.The bold values mean *p* <0.05.

### 3.3 Linear relationship between obesity indicators and retinopathy

**Figure 2:**
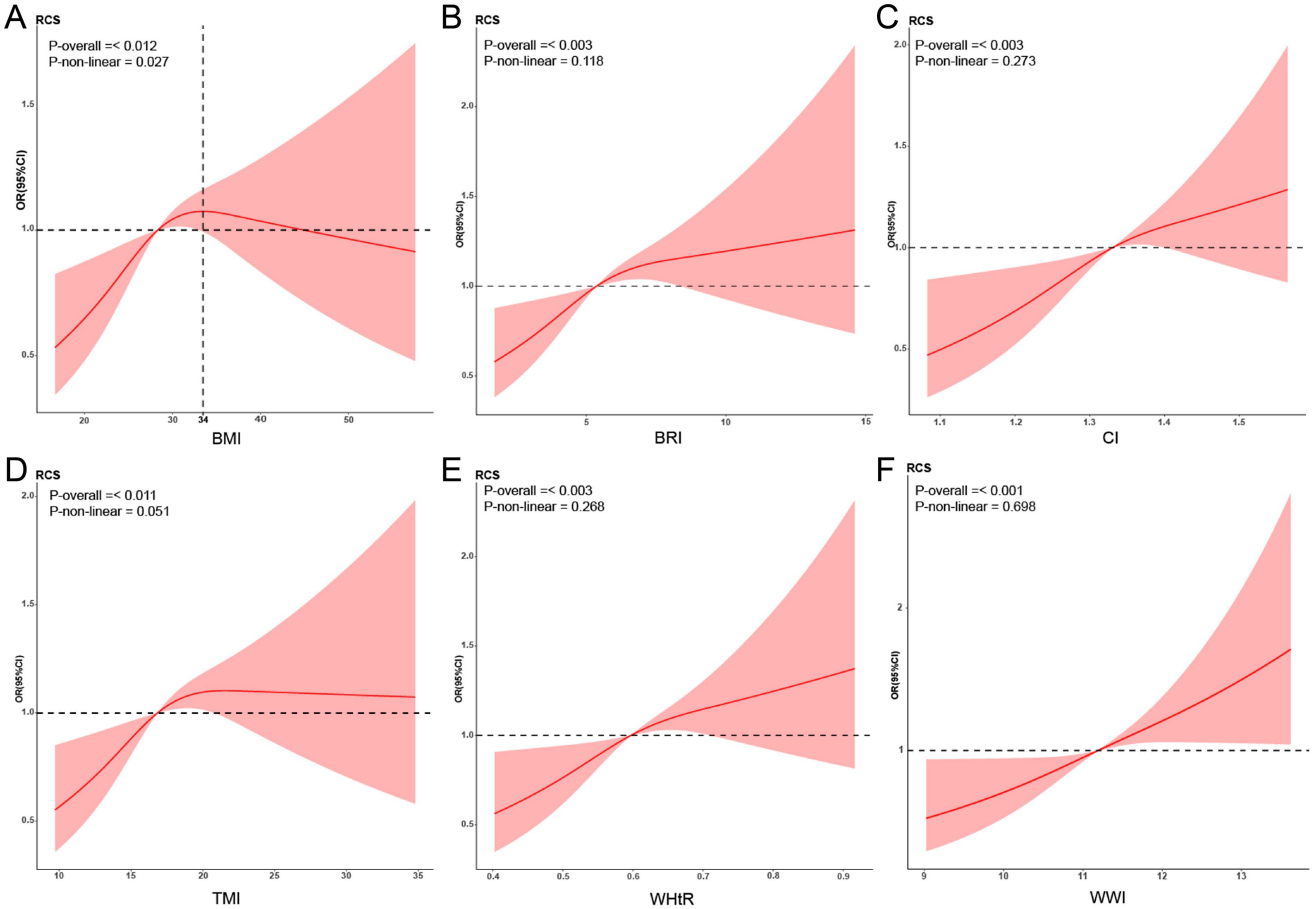
Restricted cubic spline (RCS) relationship between new obesity indicators and retinopathy. RCS analysis results for obesity indicators and retinopathy (**A**: BMI; **B**: BRI; **C**: CI; **D**: TMI; **E**: WHtR; **F**: WWI). The red curve in the figure represents the ratio (OR), and the shaded area represents the 95% confidence interval (CI). The adjustment factors include sex, age, ethnicity, educational level, marital status, economic level, alcohol consumption, smoking status, and hypertension. BMI, Body Mass Index; BRI, Body Roundness Index; CI, Conicity Index; WHtR, Waist-to-Height Ratio; TMI, Tri-ponderal Mass Index; WWI, Weight-adjusted-Waist Index.

We used RCS to test whether there was a linear relationship between the new obesity indicators and retinopathy (Figure 2). After adjusting related variables, the linear relationship between BRI, CI, WHtR, TMI, WWI, and retinopathy is significant (*p*-non-linear>0.05). The relationship between BMI and retinopathy was an inverted U-shape, with the highest point corresponding to a BMI of approximately 34 kg/m^2^.

### 3.4 Subgroup analyses

**Figure 3:**
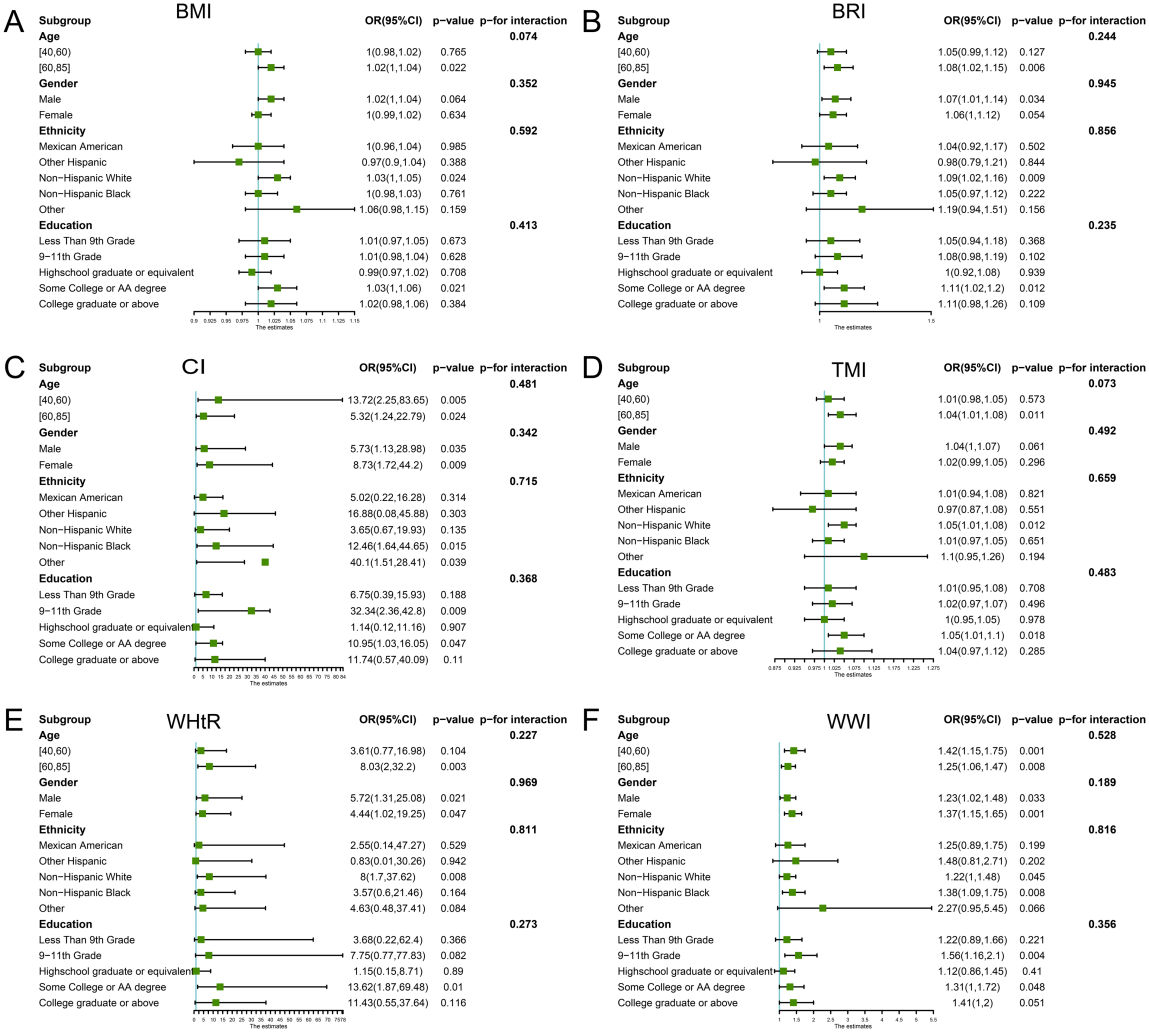
Subgroup analysis. Subgroup and interaction analyses of the association of obesity indicators and retinopathy (**A**: BMI; **B**: BRI; **C**: CI; **D**: TMI; **E**: WHtR; **F**: WWI). The green dots represent odd ratios (OR), and the bars on either side represent the 95% confidence intervals (CI). Each stratification was adjusted for sex, age, ethnicity, educational level, marital status, economic level, alcohol consumption, smoking status, and hypertension. BMI, Body Mass Index; BRI, Body Roundness Index; CI, Conicity Index; WHtR, Waist-to-Height Ratio; TMI, Tri-ponderal Mass Index; WWI, Weight-adjusted-Waist Index.

We examined the association between the obesity measures and retinopathy in different subgroups of age, sex, ethnicity, and education (Figure 3). The results showed that the obesity indicators were all statistically significant in those aged 60–85. However, the *p*-value for the interaction between the subgroups was more substantial than 0.05. This suggests that the association between the new measures and retinopathy is stable and not confounded by these covariates.

### 3.5 ROC analysis

**Figure 4:**
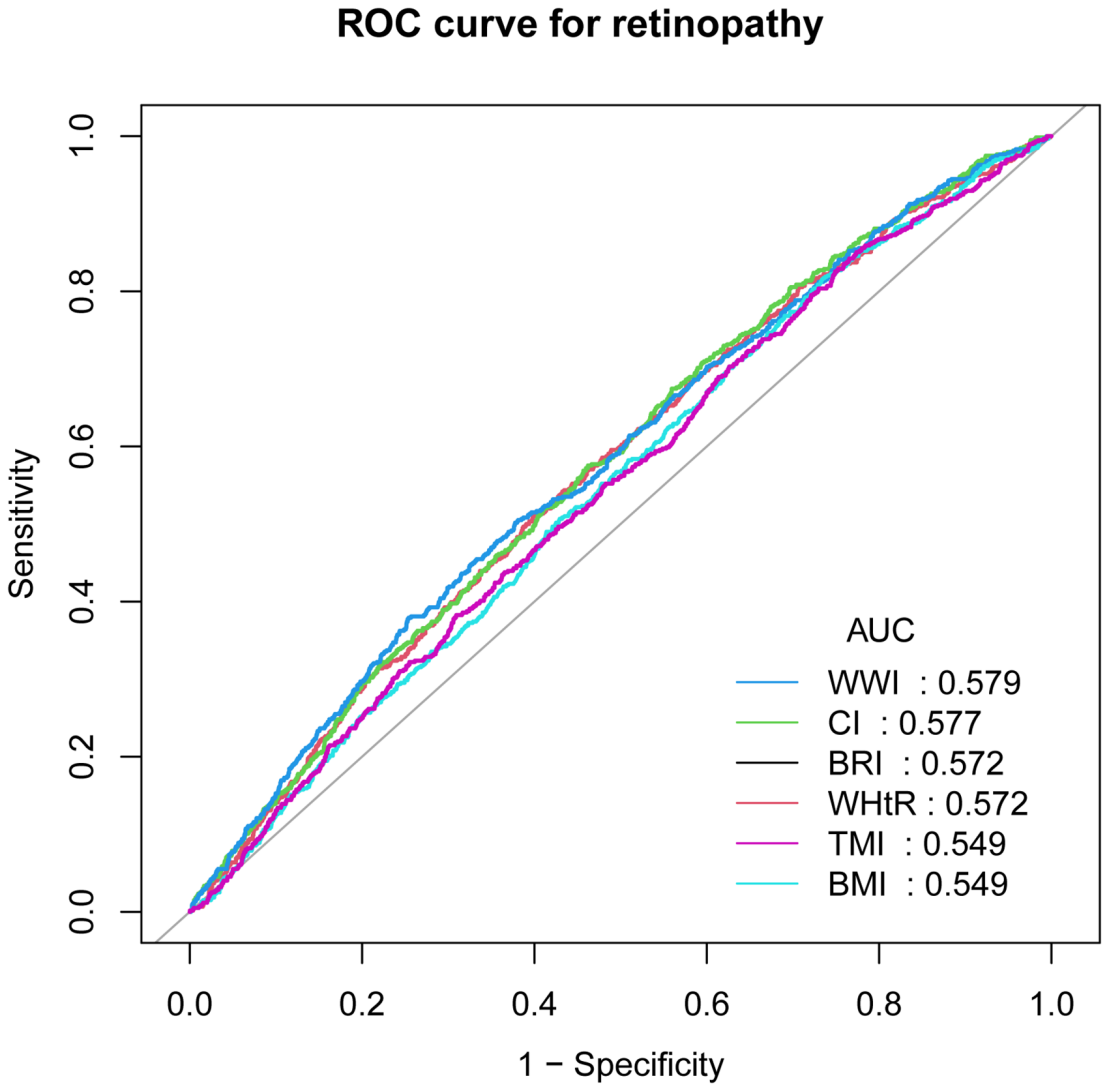
Receiver operating characteristic (ROC) curves analysis. ROC, receiver operating characteristic; AUC, area under the curve; BMI, Body Mass Index; BRI, Body Roundness Index; CI, Conicity Index; WHtR, Waist-to-Height Ratio; TMI, Tri-ponderal Mass Index; WWI, Weight-adjusted-Waist Index.

To assess the predictive value of the new measures for retinopathy, we performed ROC analysis and calculated the area under the curve (AUC) for BRI (0.572), CI (0.577), WHtR (0.572), TMI (0.549), WWI (0.579) and BMI (0.549). Finally, WWI was found to have the highest predictive value for retinopathy (see Figure 4) for details.

## 4 Discussion

This study is the first to examine the relationship between obesity indicators and retinopathy using data from 4,867 participants in the NHANES database. After adjustment for relevant covariates, BRI, CI, WHtR, TMI, and WWI were positively correlated with retinopathy. Subgroup analyses showed that this association was stable across populations. BRI, CI, WHtR, and WWI were more predictive of retinopathy than BMI, a conventional measure of obesity.

Obesity refutation has been shown to exist in many chronic diseases, and previous studies have shown that overweight and obese individuals have reduced mortality in patients with coronary heart disease, heart failure, and some types of breast cancer (26, 27). In the present study, we found an ‘obesity refutation’ of the relationship between BMI and retinopathy, and the risk of retinopathy decreases with an increase in BMI above 34 kg/m^2^. This may be related to lipocalin, secreted by white fat cells in obese people. Studies have shown that lipocalin can have anti-inflammatory and antioxidant effects under certain conditions (28, 29). Secondly, obese people are more likely to consume vitamins and minerals, nutrients that can reduce oxidative stress throughout the body and reduce retinal damage (30, 31).

Weight-adjusted-waist index is a new indicator of obesity, as proposed by Park et al. (32) A retrospective study found that the likelihood of having diabetic retinopathy decreased by 31% for every additional unit of WWI (33). Another study found a negative correlation between WWI and diabetic nephropathy when WWI < 11.48 (34). Similarly, we found that people with diabetes with WWI between 11.619 and 12.065 were less likely to develop retinopathy compared to people with diabetes with WWI (9.381–11.136). This may be related to the high levels of C-peptide in obese people with diabetes (35), which has been shown to increase retinal vascular blood flow and improve retinal endothelial cell function (36, 37). It may also counteract the production of ROS and inhibit the production of VEGF (38).

Sun et al. (39) found that WWI was more predictive of diabetes mellitus and hypertension than BMI (40). Several retrospective studies have also shown that newer indices like WWI and visceral adiposity index (VAI) are more predictive of diabetic retinopathy (41, 42). This suggests that BMI, which reflects generalized obesity, has limitations and that indicators reflecting fat distribution have more excellent clinical predictive value. Lee et al. (43) suggested that excess abdominal and visceral fat may be an essential risk factor for diabetic retinopathy. However, the above studies did not focus on fat distribution and other retinopathies. Therefore, we examined the association of these obesity markers with retinopathy in the American population. They were helpful as indicators for assessing the risk of developing retinopathy.

Obesity as a risk factor for retinopathy may be related to several aspects. Obesity is often associated with insulin resistance (44, 45), producing hyperglycemia. Prolonged hyperglycemia damages retinal endothelial cells, triggering retinal vascular hyperglycemia and leakage (46, 47), resulting in retinal damage. Second, visceral fat accumulation can disrupt the function of pancreatic β-cells, leading to persistent hyperglycemia. On the other hand, visceral fat can inhibit the secretion of adipokines (48, 49), leading to increased expression of inflammatory factors, including TNF-1A and IL-6, which damages the retina (50).

Our study is the first to comprehensively examine the association between six anthropometric measures and the prevalence of retinal disease, while also comparing their predictive value. The findings suggest that controlling obesity and improving body composition may help reduce the prevalence of retinal disorders. Among the indicators evaluated, the WWI demonstrated the strongest predictive potential. Therefore, individuals with higher WWI values may benefit from further retinal examinations. Such an approach could facilitate earlier detection and treatment of retinal disease, reduce the risk of vision loss or blindness, and ultimately conserve medical resources and alleviate societal burden. Moreover, given the close relationship between retinal health and chronic conditions such as diabetes and hypertension, paying closer attention to the retinal status of obese individuals may also contribute to improving their overall health outcomes.

We note that the study still has limitations: the population was from the United States, and the universality of these findings needs further exploration. Due to the limitations of a cross-sectional study, it was impossible to explain the specific mechanisms linking the measurements to retinopathy. In addition, it was impossible to fully adjust for relevant covariates and exclude the influence of confounding factors.

## 5 Conclusion

In this study, using NHANES data, we found that obesity indicators (BRI, CI, WHtR, TMI, WWI, and BMI) and retinopathy were significantly and positively correlated and had some predictive value for retinopathy. It is recommended that the risk of retinopathy be reduced by keeping these indicators within safe limits through a healthy lifestyle. Combining these indicators by clinicians is helpful in the prevention and early diagnosis of retinopathy.

## Data Availability

Publicly available datasets were analyzed in this study. This data can be found at: NHANES is a public database, and all researchers can access the data from www.cdc.gov/nchs/nhanes.
