# Supplementary material for: Association between obesity indicators and retinopathy in US adults: NHANES 2005–2008
Source: Front Nutr. 2025 Jun 23;12:1598240. doi: 10.3389/fnut.2025.1598240 (PMC12229866; doi:10.3389/fnut.2025.1598240)
Supplement: Supplementary file 1 [file Table_1.docx]

Table S1. Specific values of each variable by percentile

| Variables | American people | | | | Diabetic people | | | |
| --- | --- | --- | --- | --- | --- | --- | --- | --- |
|  | Q1 | Q2 | Q3 | Q4 | Q1 | Q2 | Q3 | Q4 |
| ABSI | 0.061-0.080 | 0.080-0.083 | 0.083-0.086 | 0.086-0.117 | 0.061-0.081 | 0.081-0.084 | 0.084-0.087 | 0.087-0.103 |
| BRI | 1.070-4.173 | 4.173-5.366 | 5.366-6.821 | 6.823-18.137 | 1.672-5.295 | 5.297-6.658 | 6.659-8.186 | 8.189-15.596 |
| CI | 0.974-1.271 | 1.271-1.331 | 1.331-1.387 | 1.387-1.906 | 1.120-1.319 | 1.320-1.374 | 1.374-1.423 | 1.423-1.704 |
| WHtR | 0.357-0.541 | 0.541-0.597 | 0.597-0.658 | 0.658-1.010 | 0.400-0.594 | 0.594-0.652 | 0.652-0.711 | 0.711-0.944 |
| WWI | 8.594-10.675 | 10.675-11.189 | 11.190-11.706 | 11.707-15.704 | 9.381-11.136 | 11.138-11.618 | 11.619-12.065 | 12.066-15.388 |
| BMI | 13.36-24.88 | 24.89-28.30 | 28.31-32.37 | 32.38-66.16 | 16.97-27.27 | 27.28 - 31.19 | 31.2 - 35.91 | 35.92 - 64.8 |
| TMI | 7.869-14.715 | 14.717-16.842 | 16.846-19.574 | 19.577-42.295 | 10.045-16.409 | 16.413-18.840 | 18.867-21.713 | 21.713-42.295 |
